# Editorial: Pathophysiology of Rare Hemolytic Anemias

**DOI:** 10.3389/fphys.2020.601746

**Published:** 2020-10-22

**Authors:** Richard van Wijk, Paola Bianchi

**Affiliations:** ^1^Central Diagnostic Laboratory - Research, University Medical Center Utrecht, Utrecht University, Utrecht, Netherlands; ^2^Hematology Unit, Phatophysiology of Anemias Unit, Fondazione IRCCS Ca' Granda Ospedale Maggiore Policlinico, Milan, Italy

**Keywords:** red blood cell, hemolytic anemia, enzymopathies, membranopathies, congenital dyserythropoetic anemia

The current Research Topic focuses on rare hereditary hemolytic anemias, and arises from the need to increase awareness for this heterogeneous group of disorders. Rare hereditary hemolytic anemias can be roughly categorized into hemoglobinopathies, membrane- and hydration disorders, metabolic disorders (all hyper-regenerative anemias), or defects of red blood cell production (hypo-regenerative anemias). In many cases, disease pathophysiology and genotype-to-phenotype correlations is poorly understood, complicating recognition and diagnosis, as well as development of effective treatment. This Research Topic brings together a number of studies that contribute to increasing our knowledge on the pathophysiology of red blood cell disorders, and that may raise an interest of pharmaceutical companies and device manufacturers in developing specific drugs and technological devices.

Red blood cell membrane- and hydration defects generally associated with morphological changes. However, more definitive diagnosis requires the use of additional diagnostic tests. Zaidi et al. report on their many years of experience using osmotic gradient ektacytometry, a highly specialized technique, in comparison with the eosin maleimide (EMA) binding test. The former technique provides a fluid, physiological and hence functional test of red blood cell deformability whereas the EMA binding test is a static test that provides a quantifiable measurement of the major red blood cell membrane protein band 3. Using patients with various disorders of the red cell membrane, such as hereditary spherocytosis (HS), hereditary elliptocytosis, and south-east asian ovalocytosis the authors describe the key differences between the two tests. They outline a number of pitfalls of in particular the EMA binding test but conclude that when combined both rapid tests are complimentary, and therefore worthwhile in the diagnosis of red blood cell membrane- and hydration disorders (Zaidi et al.).

Analysis of peripheral blood film is also an essential tool to evaluate red blood cell morphology. It needs to be evaluated on fresh blood. The conservation of original red blood cell shapes, obtained following glutaraldehyde fixation for imaging and in particular 3D-analysis, is a common desire. In a very punctual analysis, Abay et al. investigated and documents the subtle use of glutaraldehyde on healthy and pathologic red blood cells, and how to deal with or circumvent pitfalls.

Most red blood cell membrane disorders are inherited in an autosomal dominant manner. However, an important subgroup is autosomal recessive HS. This disease is mainly caused by bi-allelic mutations of *SPTA1*, the gene that codes for α-spectrin. Chonat et al. focus on this specific patient category and systematically compare genetic, rheological, and protein data to the clinical presentation of patients. They show that α-spectrin levels correlate to disease severity and, importantly, the obtained genetic and phenotypic data they collected shows differences in response to splenectomy, a commonly used procedure to treat patients with HS. Although the number of patients in this study was limited such results will be of importance for clinical decision making.

With specific focus on deciphering disease pathophysiology of rare hereditary hemolytic anemias in general Petkova-Kirova et al. investigated if changes in membrane conductance could be a factor contributing to phenotypic expression by means of altering cation content and subsequent disrupting volume homeostasis. They performed whole-cell patch-clamp measurements on red blood cells from patients with a variety of different rare hereditary anemias. They show that red blood cells from patients with specific molecular defects causing HS or hereditary xerocytosis (HX) show altered membrane conductance. At the same time such changes were not detected in β-thalassemia patients and patients with enzyme deficiencies such as glucose-6-phosphate dehydrogenase deficiency. Their results open up an exciting new area of research to identify the channel(s) underlying the observed changes conductances.

One red blood cell ion channel that is currently the topic of intensive investigation is *PIEZO1*. Upon mutation this mechanosensitive cation channel is associated with distinct abnormalities, either autosomal dominant HX (a rare red cell hydration disorder) due to gain of function mutations in *PIEZO1* or autosomal recessive lymphatic dysplasia with non-immune hydrops fetalis due to loss of function mutations. Andolfo et al. now for the first time report on the red blood cell changes of a case of lymphatic dysplasia. Red blood cells from this patient showed altered hydration and intracellular loss of potassium, as well as structural abnormalities such as spherocytes and stomatocytes, indicating shared features of both HS and HX. Thus, their findings complement the clinical features of mutant PIEZO1-mediated lymphatic dysplasia.

Apart from glucose-6-phosphate dehydrogenase-deficiency, enzyme deficiencies of the red blood cell are very rare metabolic disorders. Fermo et al. report here on a relatively large cohort of 12 patients with chronic hemolytic anemia due to glucose-6-phosphate isomerase (GPI) deficiency, a glycolytic enzymopathy. They describe the clinical, hematological and molecular characteristics of these patients whom they had the unique opportunity to follow from infancy to adulthood. This allowed them to report three features associated with GPI-deficiency not previously reported: (1) increased susceptibility to infections; (2) poor response to splenectomy; (3) a tendency for reticulocyte numbers to increase post-splenectomy. In addition, they show that GPI-deficient red blood cells behave differently when analyzed with osmotic gradient ektacytometry. Altogether, these findings contribute to a better understanding of disease pathophysiology and diagnosis of this rare red blood cell enzymopathy.

Sickle cell disease (SCA) is a hemoglobinopathy and a relatively simple monogenetic disorder. Yet, it's pathophysiology is highly complex and the disease truly is a multi-system disorder. Using a mouse model of SCA Charrin et al. have investigated the contribution of advanced glycation end products (AGE) and their receptor (RAGE) in the development of kidney complications, one of many complications associated with SCA. They particularly focused on the effects of RAGE inhibition by a specific RAGE-antagonist (RAP). They show that specific inhibition of RAGE blunts anemia-related markers. In addition, RAP-treatment reduced glomerular hypertrophy, interstitial fibrosis, and tubular iron deposits, possibly mediated by reduced oxidative stress markers and decreased pro-inflammatory molecule expression. Thus, this work provides proof for RAGE as an important pathogenic factor in the development of renal changes in SCA.

Another very heterogeneous group of rare disorders includes congenital dyserythropoietic anemias (CDA), that are characterized by ineffective erythropoiesis, moderate to severe anemia, distinct morphological features in bone marrow late erythroblasts, and development of secondary iron overload. The prevalence of these disorders is still not well-defined. The morphological classification, initially proposed, is now supported by the identification of the molecular lesions. Most of the causative genes involved in the pathogenesis of CDAs (*CDAN1*, and *C15orf41* for CDA-I, *SEC23B* for CDA-II or *KIF23* responsible for CDA-III) seems to be directly or indirectly involved in the erythroid maturation process or a mitotic kinesin crucial for cytokinesis.

One of the clear advantages of next generation sequencing technologies is the availability of molecular testing for rare diseases in many laboratories, resulting in the increased awareness of rare congenital conditions. On the other hand, the huge amount of data obtained should be always supported by functional studies. This is illustrated by two new variants that Russo et al. detected in the *C15orf41* gene (p. (His230Pro) and p. (Glu94Ser)) that cause a reduction of gene expression and protein production, both resulting in impaired erythroid maturation and suggesting a block of cell cycle dynamics as a putative pathogenic mechanism for *C15orf41*-related CDA-I. More recently, mutations in genes codifying for erythroid-specific transcription factors (*GATA1* and *KLF1*) have been also described in patients with dyserythropoietic anemia.

To support the clinical management and diagnostic iter of these disorders a new telemedicine tool was developed by Tornador et al., hosted on a webpage named CoDysAn and available at http://www.codysan.eu. CoDysAn algorithm was validated on 24 patients genetically diagnosed of different types of CDA and with a set of 19 hemolytic patients non-CDA-I.

The advent of new technological approaches also enabled a better knowledge of the ontogeny of erythropoiesis supporting the concept that hematopoietic stem cell themselves are highly heterogeneous and lineages separate earlier than previously thought. The coordination of these events is orchestrated by transcription factors that work in a combinatorial manner to activate and/or repress their target genes as reviewed by Barbarani et al.. The examples of GATA1 and KLF1 presented in this review suggest that in the next few years the number of mutations identified in transcription factors associated with diserythropoietic disorders will further increase.

Other less known signaling pathways seem to influence the erythroid progenitor cells in the bone marrow. Fibroblast Growth Factor 23 (FGF23) is a hormone involved in phosphate, vitamin D metabolism and has been recognized as an important regulator of bone mineralization. Erythroid progenitors highly express FGF23 protein and carry the FGF23 receptor. van Vuren et al. describe how the EPO-FGF23 pathway seems to interact with red blood cell production and the responses to endogenous erythropoietin in rare hereditary anemias, thereby providing a novel pathophysiological feature that may be common to many different forms of (rare) anemia.

Among the group of acquired hemolytic anemias paroxysmal nocturnal hemoglobinuria definitely represents a fascinating model among red blood cell diseases. Due to the lack of membrane GPI-anchored proteins, complement is strongly activated causing complement-mediated lysis. This results not only in anemia, but also in the release of free hemoglobin and iron, which catalyze the generation of reactive oxygen species and subsequent NO depletion and vasoconstriction. For untreated patients, thrombosis is the most common cause of death. To further investigate these aspects, microvesicles formation was considered by Freitas Leal et al.. The absence of GPI-anchored membrane proteins seems to both strongly increase microvesicle formation and to affects the composition of red blood cell-derived as well as platelet-derived vesicles. These data open the way to new aspect in pathophysiological mechanism of paroxysmal nocturnal hemoglobinuria, and possibly contribute to the development of new treatment strategies.

The articles collected in the present Research Topic reflect current and future directions of research, development of diagnostic tools and therapies for red blood cell related diseases. Given the rarity and the heterogeneity of this group of disorders, research, and diagnostics often merge into each other, especially for rare or undiagnosed cases. It is also evident that increasing awareness, developing of new diagnostic devices and networking activities to join expertise is crucial in this field, as highlighted recently by Kaestner and Bianchi.

## Author Contributions

RW and PB wrote the manuscript. All authors contributed to the article and approved the submitted version.

## Conflict of Interest

The authors declare that the research was conducted in the absence of any commercial or financial relationships that could be construed as a potential conflict of interest.

